# Academic entitlement and student well-being: The mediating role of the impostor phenomenon

**DOI:** 10.1371/journal.pone.0362076

**Published:** 2026-11-04

**Authors:** Amal Mubarak Alsuwaylim, Bandar A. Alsharif, Mohamed Sayed Abdellatif, Mohammed Rahmath, Ashraf Ragab Ibrahim, Mohamed Ali Nemtallah

**Affiliations:** 1 Department of Psychology, College of Education, Prince Sattam Bin Abdulaziz University, Al-Kharj, Saudi Arabia; 2 Department of Education, College of Arabic Language and Humanities Studies, Islamic University of Madinah, Madinah, Saudi Arabia; 3 Department of Computer Engineering and Information, College of Engineering in Al-Kharj, Prince Sattam Bin Abdulaziz University, Al-Kharj, Saudi Arabia; 4 Educational Psychology and Statistics Department, Faculty of Education, Al-Azhar University, Dakahlia, Egypt; University of Montenegro: Univerzitet Crne Gore, MONTENEGRO

## Abstract

Despite growing evidence linking academic entitlement to diminished student outcomes, the psychological mechanisms through which entitled academic expectations erode student well-being remain insufficiently understood. The present study examines the mediating role of the impostor phenomenon in the relationship between academic entitlement and well-being among university students. A cross-sectional correlational design was employed with a sample of 864 undergraduate students recruited from Al-Azhar University and Kafr El-Sheikh University in Egypt. Participants completed the Academic Entitlement Scale (AES-28), the Short Clance Impostor Phenomenon Scale (CIPS-10), and the BBC Well-Being Scale (BBC-WBS). Mediation analysis was conducted using the PROCESS macro with bootstrapping based on 5,000 resamples. Results revealed that academic entitlement significantly and positively predicted the impostor phenomenon, which in turn significantly and negatively predicted student well-being. The impostor phenomenon partially mediated the relationship between academic entitlement and well-being, accounting for approximately 23% of the total effect. These findings suggest that entitlement-driven expectations are associated with lower overall well-being (composite score), both directly and indirectly by fostering fraudulence-related self-doubt. Mediation was not tested separately for psychological, physical, and relational well-being. Universities, particularly in comparable Egyptian and collectivist contexts, may consider addressing both entitled academic attitudes and impostor-related cognitions through counseling interventions and pedagogical strategies.

## Introduction

Modern universities increasingly face a critical challenge wherein students’ expectations of academic success diverge sharply from their actual psychological well-being, necessitating urgent examination of both behavioral and internal coping mechanisms that mediate this gap [1–8]. Students entering higher education commonly carry aspirations of achievement, belonging, and career advancement; however, the compounding pressures of academic workload, life transitions, financial strain, and competitive environments frequently erode psychological functioning [9–14]. This misalignment between anticipated success and experienced well-being underscores the necessity of investigating the internal psychological resources and adaptive coping strategies that enable students to persist and thrive [15].

Undergraduate university students represent the target population of this research, a demographic uniquely vulnerable to psychological struggles due to the convergence of intense academic pressure, identity evaluation, and heightened risks of mental health decline [16–21]. During the undergraduate years, students navigate simultaneous demands of academic performance, emerging adulthood, and social role adjustment, all of which amplify psychological distress and self-evaluative processes [22–24]. Evidence consistently demonstrates that this population exhibits elevated prevalence rates of depression, anxiety, and psychological distress compared to age-matched peers, with vulnerabilities further compounded by financial strain, social isolation, and inadequate access to mental health support [25–27].

Academic entitlement, defined as students’ tendency to expect high grades and special rewards irrespective of their actual effort or performance, has emerged as a significant concern in higher education that warrants systematic examination [28–33]. This disposition disrupts student-teacher dynamics by fostering demands for unwarranted accommodations, grade negotiation, and unprofessional communication, thereby eroding the mutual trust essential for productive academic relationships [29,34]. Furthermore, academically entitled students demonstrate diminished classroom engagement, weakened emotion regulation, and greater tolerance for academic dishonesty, collectively establishing unrealistic standards that compromise students’ motivation, self-regulated learning, and long-term professional development [30,31,35].

Within the university context, student well-being is understood as a multidimensional construct encompassing both subjective happiness — including life satisfaction and positive affect — and psychological functioning, comprising self-acceptance, autonomy, purpose, and environmental mastery [16,25,36–38]. Both dimensions are severely strained when students encounter academic setbacks or harbor unrealistic self-perceptions, as harsh self-evaluation and inflated personal demands intensify distress, undermine confidence, and impair adaptive coping [19,39,40]. Research further indicates that self-inflicted stress rooted in distorted competence beliefs erodes students’ sense of belonging and academic adjustment, establishing a compelling rationale for investigating how unrealistic academic self-perceptions — such as entitlement — intersect with well-being outcomes [8,41].

Central to the present study is the impostor phenomenon, defined as a persistent internal experience of intellectual phoniness whereby individuals feel fraudulent and undeserving of their achievements despite clear objective evidence of competence and success [42–46]. This study proposes that the impostor phenomenon functions as a critical explanatory bridge between academic entitlement and diminished well-being, whereby students’ externally expressed entitlement beliefs paradoxically coexist with, and may provoke, deep-seated self-doubt and fears of exposure [47–49]. Such fraudulence-related cognitions have been consistently linked to anxiety, reduced self-efficacy, and impaired psychological functioning, rendering the impostor phenomenon a theoretically compelling mediator through which entitlement behaviors translate into poorer well-being outcomes [50,51].

A critical psychological conflict emerges when students’ external academic entitlement — the conviction that success is automatically deserved — collides with internal impostor feelings characterized by persistent fears of incompetence and fraudulent achievement, producing severe anxiety and markedly diminished well-being [42,46,48,52–54]. This self-discrepancy manifests as an inability to genuinely internalize accomplishments, whereby achievements are reattributed to luck or deception rather than actual ability, perpetuating dread of eventual exposure [49,55,56]. The resulting psychological friction sustains a destructive cycle of over-preparation, self-doubt, and helplessness that compounds distress, erodes self-esteem, and significantly undermines both hedonic and psychological well-being among university students [57–59].

To theoretically anchor the relationships among academic entitlement, the impostor phenomenon, and well-being, the present study adopts a student-adapted Job Demands-Resources (JD-R) framework enriched by self-concept incongruence theory, wherein entitlement-driven expectations of recognition and special academic treatment function as psychological demands, while impostor feelings represent the resulting internal identity conflict that depletes psychological resources and erodes well-being [9,43,60–62]. Within this framework, unstable success attributions and perfectionistic self-standards intensify impostorism by rendering achievements externally validated yet internally unowned, systematically exhausting students’ psychological functioning [53,56,63]. Protective resources including self-efficacy, resilience, and psychological capital buffer this depletion process, further corroborating the JD-R mechanism as the most empirically coherent theoretical lens for the present investigation [48,49,64].

Despite the growing bodies of literature independently establishing that academic entitlement associates with diminished academic performance, reduced engagement, and lower life satisfaction [31,65,66], and that the impostor phenomenon consistently predicts anxiety, depression, burnout, and compromised psychological well-being [48,49,67–69], evidence on whether the impostor phenomenon explains the pathway from entitled academic attitudes to student well-being remains scarce [43,70,71]. An association between academic entitlement and impostor feelings has been documented among graduate students [71], and other mediators of entitlement outcomes have been tested, including mindfulness, emotional intelligence, and locus of control [32,59,72]. To our knowledge, however, no study has tested the impostor phenomenon as a mediator linking academic entitlement to well-being among undergraduates, particularly in a non-Western context. The present study addresses this gap.

The present study aims to examine the relationships among academic entitlement, the impostor phenomenon, and well-being among Egyptian university students, with particular focus on testing the mediating role of the impostor phenomenon in the pathway from academic entitlement to student well-being. Specifically, the study investigates whether academic entitlement significantly and positively predicts the impostor phenomenon, whether the impostor phenomenon significantly and negatively predicts student well-being after controlling for academic entitlement, whether academic entitlement retains a significant direct negative effect on well-being after accounting for the mediator, and whether the impostor phenomenon partially mediates the negative association between academic entitlement and student well-being, such that a meaningful portion of the detrimental effect of entitlement on overall well-being (the composite score) operates through impostor-related self-doubt.

## Materials and methods

### Ethics statement

The study protocol was approved by the Research Ethics Committee of the Faculty of Education at Al-Azhar University, Egypt (Ref. No. IRB-AZHAR-EDU-2025–011). Participation was voluntary and anonymous. Informed consent was obtained electronically: before starting the Google Forms questionnaire, participants read an information sheet describing the study aims, procedures, and confidentiality, and indicated their agreement to participate before proceeding. All participants were aged 18 years or older, so parental or guardian consent was not required.

### Study design

This study employed a cross-sectional correlational design to examine the relationships among academic entitlement, the impostor phenomenon, and student well-being, and to test the mediating role of the impostor phenomenon in the pathway from academic entitlement to well-being.

### Participants

This study was conducted across six faculties at Al-Azhar University and Kafr El-Sheikh University in Egypt. Data were collected via Google Forms during March and April 2026, corresponding to the second semester of the academic year 2025/2026. Two independent samples were recruited: a psychometric sample (N = 672) and a main sample (N = 864). The mean age of the psychometric sample was 20.56 years (SD = 1.76), while the mean age of the main sample was 20.62 years (SD = 1.70), with ages ranging from 18 to 23 years across both samples. Demographic characteristics of both samples are presented in Table 1.

**Table 1:** Demographic characteristics of study participants.

Variable	Category	Psychometric Sample n	Psychometric Sample %	Main Sample n	Main Sample %
Gender	Male	236	35.11%	391	45.3%
	Female	436	64.89%	473	54.7%
Academic Year	First Year	171	25.4%	206	23.8%
	Second Year	167	24.9%	223	25.8%
	Third Year	160	23.8%	233	27.0%
	Fourth Year	174	25.9%	202	23.4%
Residence	Rural	329	49.0%	542	62.7%
	Urban	343	51.0%	322	37.3%

### Measures

The Academic Entitlement Scale (AES-28) [30] is a 28-item self-report scale developed and psychometrically validated specifically for Egyptian university students, used in the present study to assess academic entitlement. The scale comprises five dimensions: Externalized Responsibility (ER; 5 items), Entitled Expectations (EE; 5 items), Consumer Mindset (CM; 6 items), Control Over Learning Environment (COLE; 7 items), and Reward for Minimal Effort (RME; 5 items). Items are rated on a five-point Likert scale ranging from 1 (*Strongly Disagree*) to 5 (*Strongly Agree*). Confirmatory factor analysis (CFA) in the present sample demonstrated acceptable model fit: chi-square/degrees of freedom ratio (CMIN/DF) = 1.935, root mean square residual (RMR) =  .020, goodness-of-fit index (GFI) =  .916, adjusted GFI (AGFI) =  .901, comparative fit index (CFI) =  .951, and root mean square error of approximation (RMSEA) =  .037. Internal consistency reliability was strong across all subscales and at the total scale level, with omega coefficients (ω) ranging from  .759 (ER) to  .872 (COLE), and a total scale ω = .912.

The Short Clance Impostor Phenomenon Scale (CIPS-10) [73] is a 10-item abbreviated version of the original 20-item Clance Impostor Phenomenon Scale, used here to measure the impostor phenomenon. The scale assesses individuals’ tendency to doubt their achievements and to fear exposure as incompetent despite clear evidence of success. Although the scale has theoretical multidimensional underpinnings, factor analytic evidence supports its use as a unidimensional measure, with the total score being the most reliable index for interpretation. Items are rated on a seven-point frequency scale ranging from 1 (*never*) to 7 (*always*). CFA results indicated excellent model fit: CMIN/DF = 1.218, RMR = .025, GFI = .989, AGFI = .980, CFI = .997, and RMSEA = .018. The scale demonstrated good internal consistency (ω = .816).

The BBC Well-Being Scale (BBC-WBS) [74] is a 24-item instrument used to assess student well-being across three dimensions: Psychological Well-Being (PWB; 12 items), Physical Health and Well-Being (PHWB; 7 items), and Relationships (REL; 5 items). Items are rated on a four-point scale with response options of “not at all,” “a little,” “very much,” and “extremely.” The CIPS-10 and BBC-WBS were translated from English into Arabic (the participants’ native language) and back-translated by three independent bilingual experts to ensure linguistic equivalence. CFA supported adequate model fit: CMIN/DF = 1.429, RMR = .012, GFI = .959, AGFI = .950, CFI = .981, and RMSEA = .025. Internal consistency was satisfactory across subscales (ω ranging from  .716 for REL to  .888 for PWB) and at the total scale level (ω = .895).

Regarding cultural validity, the AES-28 was developed and validated specifically for Egyptian university students, whereas the CIPS-10 and BBC-WBS were translated into Arabic, back-translated, and evaluated using confirmatory factor analysis and reliability analyses in the present data. Measurement invariance across gender, residence, academic year, or institution was not tested, and this is acknowledged as a limitation.

### Data analysis

Data were analyzed using IBM SPSS Statistics version 27 and AMOS version 26. Descriptive statistics and bivariate correlations were computed to characterize study variables and examine their pairwise associations. A common method bias test was conducted using exploratory factor analysis, with results indicating that a single factor accounted for 29.58% of total variance, falling below the 50% threshold and suggesting that common method variance did not pose a significant threat to the validity of findings. Mediation analysis was performed using the PROCESS macro (Model 4), with bootstrapping based on 5,000 resamples and 95% bias-corrected confidence intervals to test the indirect effect of academic entitlement (X) on well-being (Y) through the impostor phenomenon (M).

## Results

### Preliminary analyses

Descriptive statistics for all study variables are presented in Table 2. Academic entitlement total scores (AES-28) ranged from 28 to 121 (M = 82.31, SD = 14.28), indicating moderate-to-high levels of entitlement in the sample. Impostor phenomenon scores (CIPS-10) ranged from 10 to 61 (M = 36.76, SD = 7.72), and well-being total scores (BBC-WBS) ranged from 36 to 96 (M = 61.19, SD = 9.40). Among the AES-28 subscales, the COLE dimension yielded the highest mean (M = 20.63, SD = 4.30), while the ER and EE dimensions yielded the lowest and most comparable means (M = 14.62, SD = 3.11 and 3.08, respectively).

**Table 2:** Descriptive statistics for study variables.

Variable	Min	Max	M	SD	Variance
1. ER	5.00	24.00	14.62	3.11	9.67
2. EE	5.00	24.00	14.62	3.08	9.52
3. CM	6.00	29.00	17.29	3.56	12.68
4. COLE	7.00	32.00	20.63	4.30	18.52
5. RME	5.00	24.00	15.15	3.09	9.52
6. AES-28	28.00	121.00	82.31	14.28	204.04
7. CIPS-10	10.00	61.00	36.76	7.72	59.56
8. PWB	17.00	48.00	30.03	5.27	27.80
9. PHWB	8.00	32.00	20.50	3.63	13.21
10. REL	6.00	16.00	10.66	1.80	3.24
11. BBC-WBS	36.00	96.00	61.19	9.40	88.29

Note. M = mean; SD = standard deviation. ER = Externalized Responsibility; EE = Entitled Expectations; CM = Consumer Mindset; COLE = Control Over Learning Environment; RME = Reward for Minimal Effort; AES-28 = Academic Entitlement Scale total score; CIPS-10 = Short Clance Impostor Phenomenon Scale total score; PWB = Psychological Well-Being; PHWB = Physical Health and Well-Being; REL = Relationships; BBC-WBS = BBC Well-Being Scale total score.

### Bivariate correlations

Bivariate Pearson correlation coefficients among all study variables are reported in Table 3. Academic entitlement (AES-28) was significantly and positively correlated with the impostor phenomenon (CIPS-10; r = .413, p < .001), and significantly and negatively correlated with well-being total scores (BBC-WBS; r = −.475, p < .001). The CIPS-10 was also negatively correlated with BBC-WBS (r = −.416, p < .001). All five AES-28 subscales demonstrated significant positive correlations with the CIPS-10 (ranging from r = .322 to  .361) and significant negative correlations with all three BBC-WBS subscales (ranging from r = −.309 to −.390), providing preliminary support for the hypothesized directional relationships. Furthermore, a common method bias test using exploratory factor analysis revealed that a single factor accounted for 29.58% of the total variance, well below the 50% threshold, suggesting that common method variance was unlikely to substantially distort the findings.

**Table 3:** Pearson correlation matrix among study variables.

Variable	1	2	3	4	5	6	7	8	9	10	11
1. ER	1										
2. EE	.601**	1									
3. CM	.621**	.606**	1								
4. COLE	.617**	.634**	.602**	1							
5. RME	.614**	.617**	.611**	.632**	1						
6. AES-28	.821**	.822**	.829**	.859**	.826**	1					
7. CIPS-10	.331**	.322**	.350**	.351**	.361**	.413**	1				
8. PWB	−.363**	−.333**	−.362**	−.347**	−.390**	−.430**	−.369**	1			
9. PHWB	−.329**	−.320**	−.319**	−.356**	−.369**	−.407**	−.361**	.649**	1		
10. REL	−.309**	−.330**	−.324**	−.336**	−.368**	−.400**	−.365**	.588**	.615**	1	
11. BBC-WBS	−.390**	−.374**	−.388**	−.397**	−.432**	−.475**	−.416**	.925**	.868**	.759**	1

Note. **p < .01 (two-tailed).

### Mediation analysis

To test the hypothesized mediation model, a simple mediation analysis was conducted using PROCESS Macro Model 4, with academic entitlement (AES-28) as the predictor variable (X), the impostor phenomenon (CIPS-10) as the mediator (M), and total well-being (BBC-WBS) as the outcome variable (Y). Path coefficients and model parameters are presented in Table 4, and the decomposition of total, direct, and indirect effects is presented in Table 5. The mediation model is visually depicted in Fig 1. The model was estimated once, using the composite BBC-WBS score as the outcome; separate models for the psychological, physical, and relational subscales were not estimated.

**Table 4:** Path coefficients for the mediation model.

Outcome	Predictor	β (Std.)	B (Unstd.)	SE	t	LLCI	ULCI
CIPS-10 (M)	AES-28 (X)	.413	.223	.017	13.30***	.190	.256
BBC-WBS (Y)	AES-28 (X)	−.366	−.241	.021	−11.56***	−.282	−.200
BBC-WBS (Y)	CIPS-10 (M)	−.265	−.323	.039	−8.38***	−.399	−.248

Note. Confidence intervals are bias-corrected bootstrap intervals based on 5,000 resamples. ***p < .001.

**Table 5:** Total, direct, and indirect effects of academic entitlement on well-being.

Effect Type	B	β	SE	p	95% CI [LL, UL]	% of Total
Total effect (X → Y)	−.313	−.475	.020	< .001	[-.351, -.274]	100%
Direct effect (X → Y)	−.241	−.366	.021	< .001	[-.282, -.200]	76.97%
Indirect effect (X → M → Y)	−.072	−.110	.015	---	[-.102, -.042]	23.03%

**Fig 1:**
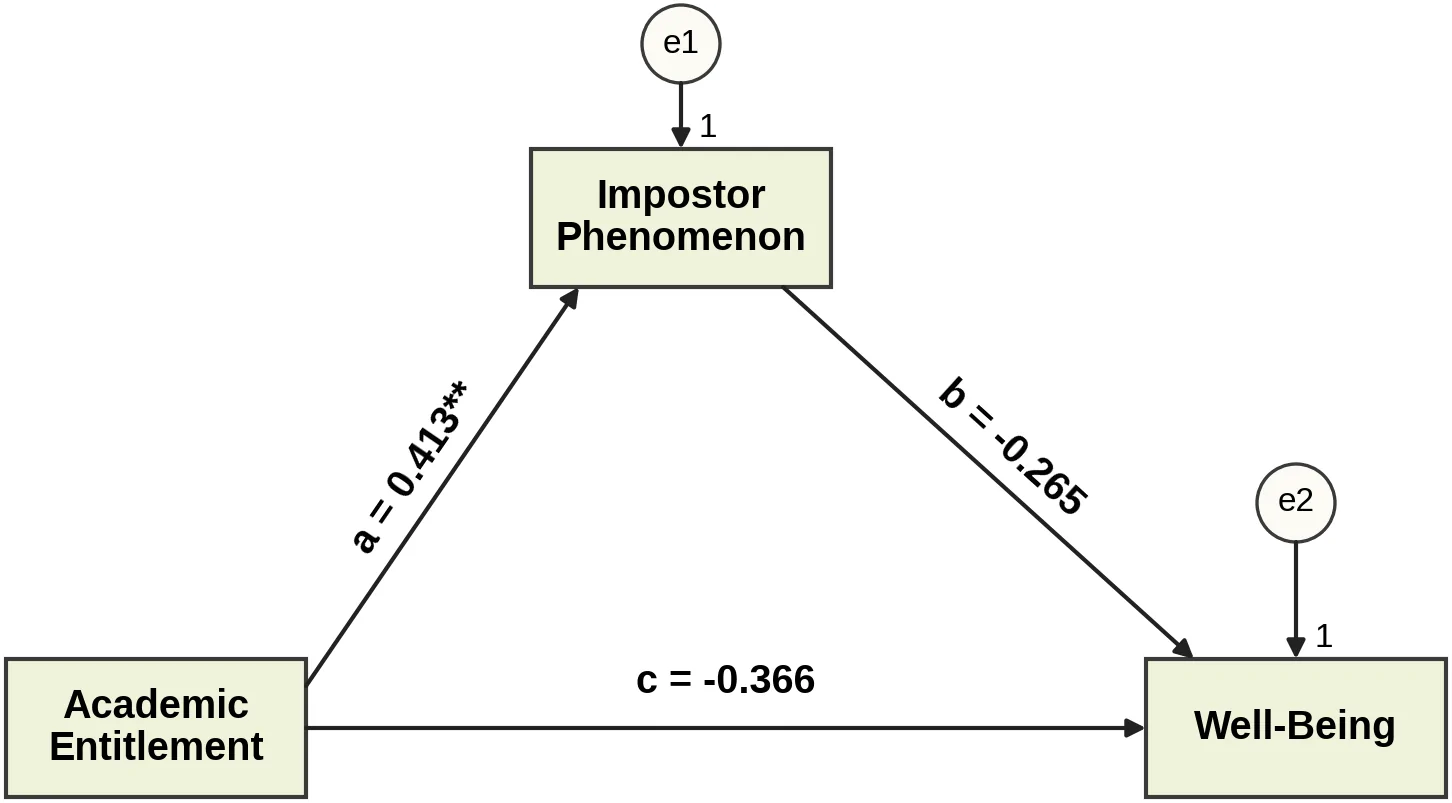
Mediation model: the impostor phenomenon as a mediator between academic entitlement and well-being. Note. Path a = effect of academic entitlement (X) on the impostor phenomenon (M); path b = effect of the impostor phenomenon on well-being (Y), controlling for X; path c′ = direct effect of X on Y, controlling for M; path c = total effect of X on Y (c = c′ + a × b). Arrows indicate the hypothesized direction of effects. Values are standardized coefficients; **p < .001. Total effect c = −.475; indirect effect a × b = −.110 (unstandardized B = −.072, 95% bootstrap CI [−.102, − .042]).

With respect to the first path in the mediation model (path a), academic entitlement significantly and positively predicted the impostor phenomenon (β = .413, B = .223, SE = .017, t = 13.30, p < .001), accounting for 17.02% of the variance in CIPS-10 scores (R^2^ = .170). With respect to the second path (path b), the impostor phenomenon significantly and negatively predicted well-being after controlling for academic entitlement (β = −.265, B = −.323, SE = .039, t = −8.38, p < .001). Academic entitlement also retained a significant direct negative effect on well-being after accounting for the mediator (path c’: β = −.366, B = −.241, SE = .021, t = −11.56, p < .001). The full model explained 28.43% of the variance in well-being (R^2^ = .284).

The results pertaining to the decomposition of total, direct, and indirect effects are presented in Table 5. The total effect of academic entitlement on well-being was significant and negative (β = −.475, B = −.313, SE = .020, 95% CI [−.351, −.274]). The indirect effect of academic entitlement on well-being via the impostor phenomenon was also significant (β = −.110, B = −.072, SE = .015, 95% bootstrap CI [−.102, −.042]), as the confidence interval excluded zero. The indirect effect accounted for 23.03% of the total effect, whereas the direct effect remained significant and accounted for 76.97% of the total effect. These findings indicate partial mediation, whereby the impostor phenomenon partially explains the negative association between academic entitlement and student well-being.

The present study demonstrates that academic entitlement exerts a significant negative effect on student well-being, both directly and indirectly through the impostor phenomenon, which functioned as a partial mediator accounting for approximately 23% of the total effect. All bivariate correlations among academic entitlement, the impostor phenomenon, and well-being and its subscales were statistically significant in the theoretically expected directions, corroborating the coherence of the proposed conceptual model. Collectively, these findings underscore the role that impostor-related self-doubt plays in linking entitled academic expectations to lower overall well-being (composite score). Because the subscales were not modeled separately, the results do not show that the indirect effect is equivalent across psychological, physical, and relational well-being.

## Discussion

The present study examined the relationships among academic entitlement, the impostor phenomenon, and well-being in a sample of 864 Egyptian university students, with particular focus on the mediating role of the impostor phenomenon. Results confirmed that academic entitlement negatively and significantly predicted student well-being, both directly and indirectly through the impostor phenomenon, which accounted for approximately 23% of the total effect.

This pattern of partial mediation indicates that while academic entitlement is directly associated with lower overall well-being (composite score), a meaningful portion of this detrimental effect operates through the psychological mechanism of impostor-related self-doubt. Because mediation was tested only for the composite score, conclusions about specific well-being dimensions are not warranted. These findings support the theoretical framework proposed in this study and advance understanding of how entitlement-driven expectations translate into compromised student well-being.

Among the most notable findings warranting attention is the magnitude and directionality of the relationship between academic entitlement and the impostor phenomenon (β = .413). This positive association is theoretically counterintuitive, as entitlement implies an inflated sense of deserving, whereas impostorism reflects pervasive self-doubt and fear of exposure. Yet this apparent paradox aligns with the self-discrepancy framework: students who externalize responsibility, expect unearned rewards, and adopt a consumer mindset toward education may simultaneously harbor deep uncertainty about whether their perceived entitlements are genuinely merited. This internal contradiction — publicly asserting deservingness while privately doubting competence — generates precisely the fraudulence-related cognitions characteristic of the impostor phenomenon, ultimately intensifying psychological distress and eroding well-being beyond the direct effect of entitlement alone.

The finding that academic entitlement negatively predicts student well-being is consistent with prior research demonstrating that entitled academic attitudes are associated with diminished engagement, reduced life satisfaction, and increased psychological distress [31,66,70]. The positive association between academic entitlement and the impostor phenomenon builds on earlier empirical work by Gibson-Beverly and Schwartz [71], who documented links between attachment, entitlement, and impostor feelings among female graduate students; the present findings extend this evidence to Egyptian undergraduates and to well-being outcomes.

Furthermore, the established negative relationship between the impostor phenomenon and well-being corroborates a robust body of evidence documenting that impostorism predicts anxiety, depression, burnout, and impaired psychological functioning [48,49,68,69]. The partial mediation pattern observed here aligns with theoretical arguments that internal identity conflicts deplete psychological resources and erode well-being through mechanisms consistent with the Job Demands-Resources framework [43,59,61].

The cultural context may also shape these relationships. In collectivist settings such as Egypt, academic entitlement may be tied less to individual self-interest and more to family expectations, social reputation, or the high stakes attached to degree attainment. Likewise, impostor feelings may reflect fear of disappointing or shaming one's family or community rather than purely individual self-doubt. These interpretations are speculative because family expectations, shame, and cultural values were not measured here. Accordingly, the associations should be interpreted within the Egyptian university context, and cross-cultural replication is needed before they are generalized.

The findings carry meaningful implications for university practitioners, counselors, and policymakers, particularly within Egyptian and comparable collectivist higher-education contexts. Institutions in such contexts may consider developing proactive programming that challenges entitlement-oriented attitudes early in students’ academic trajectories, particularly through first-year orientation curricula emphasizing effort-based achievement and academic integrity. Such programming should be adapted to local cultural expectations and evaluated before wider adoption.

Simultaneously, given the significant role of the impostor phenomenon as a mediating mechanism, counseling services **may** integrate evidence-based interventions specifically targeting impostorism, such as cognitive restructuring, self-compassion training, and peer mentoring programs. At the instructional level, faculty awareness of how entitled expectations manifest in classroom behaviors — including grade negotiation and avoidance of responsibility — can inform more constructive pedagogical responses that redirect students toward intrinsic motivation and genuine academic engagement without inadvertently reinforcing fraudulence-related self-perceptions.

Several limitations of the present study should be acknowledged when interpreting these findings. First, the cross-sectional design precludes causal inference; longitudinal or experimental designs are required to establish temporal directionality among the three constructs. Second, data were collected exclusively from Egyptian university students at two institutions, limiting the generalizability of findings to other cultural, national, or institutional contexts (including individualistic Western settings) where academic norms and entitlement expressions may differ substantially. Third, all measures were self-reported, which introduces the possibility of response bias and socially desirable responding, particularly for sensitive constructs such as academic entitlement. Fourth, the mediation model accounted for 28.43% of variance in well-being, indicating that additional explanatory variables — such as self-efficacy, resilience, or social support — remain unexamined and may further illuminate the entitlement–well-being pathway.

Fifth, locus of control was not measured or included in the model. External locus of control is theoretically and empirically linked to both academic entitlement, as students who attribute outcomes to external forces may hold more entitled expectations, and the impostor phenomenon. Because it was not controlled, the observed associations may be partly confounded by it. Adjusting for locus of control could attenuate the paths from entitlement to impostorism and to well-being, and therefore the size of the indirect effect. Sixth, the mediation model used only the composite well-being score, so it cannot show whether the indirect effect is similar for psychological, physical, and relational well-being. Finally, although the AES-28 was validated for Egyptian students and the CIPS-10 and BBC-WBS were translated and back-translated, measurement invariance across subgroups and cultures was not established.

Future research should address the identified limitations by employing longitudinal designs to track how academic entitlement and the impostor phenomenon co-evolve across undergraduate years and how these trajectories relate to changes in student well-being over time. Investigators are encouraged to replicate and extend this model across diverse cultural and institutional contexts, including Western universities and non-STEM disciplines, to establish boundary conditions for the observed mediation effect. Additionally, future studies should examine potential moderators of the entitlement–impostorism–well-being pathway, including gender, academic year, socioeconomic status, and parenting style, each of which may differentially amplify or attenuate the mediated relationships identified here. Intervention research evaluating the effectiveness of targeted impostor phenomenon programs in reducing the psychological toll of academic entitlement represents a particularly promising and clinically relevant avenue for future inquiry. Studies should also test the model separately for each well-being dimension, include locus of control as a covariate or competing mediator, and establish measurement invariance across groups and cultures.

## Conclusion

To our knowledge, the present study is the first to test the impostor phenomenon as a mediator between academic entitlement and well-being among undergraduates in a non-Western (Egyptian) context, revealing a partial mediation pathway through which entitled academic expectations are associated with lower overall well-being both directly and indirectly by fostering fraudulence-related self-doubt. These findings enrich the theoretical understanding of how superficially contradictory constructs — external entitlement and internal self-doubt — converge to undermine students’ overall well-being, although the pattern for specific well-being dimensions remains to be tested. Practically, the results suggest that universities in Egypt and comparable contexts may benefit from addressing both the entitled attitudes students bring to higher education and the impostor-related cognitions these attitudes may paradoxically engender, with the ultimate goal of cultivating academic environments that foster genuine competence, psychological security, and sustainable well-being. Generalization beyond this cultural context requires further research.
