# Supplementary material for: Academic entitlement and student well-being: The mediating role of the impostor phenomenon
Source: PLoS One. 2026 Nov 4;21(11):e0362076. doi: 10.1371/journal.pone.0362076 (PMC13734581; doi:10.1371/journal.pone.0362076)
Supplement: Supplementary file 1 [file pone.0362076.s001.docx]

Thank you for the opportunity to review the manuscript “**Academic Entitlement and Student Well-Being: The Mediating Role of the Impostor Phenomenon**”. The topic is timely, and the Egyptian sample adds valuable non-Western evidence to the literature. Below are several comments aimed at improving the paper:

1. **The "First Empirical Evidence" Claim**

The authors assert that the present study "provides the first empirical evidence that the impostor phenomenon mediates the relationship between academic entitlement and student well-being." This is a strong novelty claim and requires careful scrutiny. The literature already documents an association between academic entitlement and the impostor phenomenon in graduate student populations, even if the full mediation model with well-being as an outcome has not been tested. The authors should clarify the scope of their literature search and, if prior related work exists, temper the claim accordingly (e.g., "first in a non-Western context" or "first to test well-being as an outcome"). As it stands, the claim of absolute priority is difficult to defend and should either be substantiated or revised.

2. "**Holistic Functioning" and the Three Dimensions of Well-Being**

The conclusion claims that academic entitlement undermines students' "psychological, physical, and relational well-being" and refers to "holistic functioning." However, it is unclear whether the mediation model was tested separately for each of the three well-being dimensions or only for a composite well-being score. If a single composite score was used, the claim that the indirect effect operates uniformly across all three dimensions is not warranted. The mediating role of the impostor phenomenon may be strong for psychological well-being but weak or non-significant for physical or relational well-being.

3. **Generalizability and Cultural Context**

The conclusion speaks of "universities" in general and calls upon them to address entitled attitudes and impostor-related cognitions. However, the sample consists exclusively of **Egyptian university students.** This raises significant concerns about generalizability. In collectivist cultures, academic entitlement may manifest differently than in individualistic Western cultures — for example, it may be tied more closely to family expectations or social reputation rather than individual self-interest. Similarly, the impostor phenomenon in collectivist contexts may be driven more by fear of bringing shame to one's family or community than by individual self-doubt. The authors should (a) acknowledge that their findings may not generalize beyond the Egyptian context, (b) discuss how cultural factors might shape the relationships observed, and (c) avoid overgeneralizing their practical recommendations to "universities" as a universal category. Ideally, the manuscript should also report whether the measurement instruments used were validated for the Egyptian context and whether measurement invariance was established.

4**. Insufficient Description of Academic Entitlement Subscales in Table 2**

In Table 2, which presents descriptive statistics, the table note does not provide any explanation of the academic entitlement subscales.

5**. Missing Path Labels in Figure 1**

In Figure 1, the labels for paths a, b, and c′ are not visible. Without these labels, the reader cannot easily map the figure onto the hypotheses stated in the introduction or the results reported in the text. This creates unnecessary confusion and undermines the clarity of the mediation model. The authors should revise Figure 1 to explicitly label all paths (a, b, c, and c′), include the direction of arrows, and ensure that the figure caption explains what each path represents. Consistency between the figure, the hypotheses, and the results tables is essential.

6. **Locus of Control as an Unconsidered Variable**

It appears that locus of control could have been a relevant variable in this study but was not included in the model. Locus of control is theoretically and empirically linked to both academic entitlement (external locus of control may foster entitled expectations) and the impostor phenomenon. At a minimum, the authors should acknowledge this limitation in the limitations section and discuss how controlling for locus of control might have altered the observed relationships.
