# Supplementary material for: Academic entitlement and student well-being: The mediating role of the impostor phenomenon
Source: PLoS One. 2026 Nov 4;21(11):e0362076. doi: 10.1371/journal.pone.0362076 (PMC13734581; doi:10.1371/journal.pone.0362076)
Supplement: Supplementary file 2 [file pone.0362076.s003.docx]

**Response to Reviewers**

Manuscript PONE-D-26-37507: "Academic Entitlement and Student Well-Being: The Mediating Role of the Impostor Phenomenon"

Dear Dr. Joksimovic and Reviewers,

Thank you for the constructive feedback. We have addressed every point below. In the "Revised Manuscript with Track Changes," all changes are marked.

# Journal Requirements

**Comment 1:** *Ensure the manuscript meets PLOS ONE style requirements, including file naming.*

**Response 1:** We revised the manuscript to meet PLOS ONE style requirements, including file naming.

**Comment 2:** *Provide details regarding participant consent.*

**Response 2:** We moved the Ethics statement to the beginning of the Methods section and expanded it. It now states that approval was granted by the Research Ethics Committee of the Faculty of Education, Al-Azhar University (Ref. No. IRB-AZHAR-EDU-2025-011), that participation was voluntary, and that informed consent was obtained electronically before participants started the Google Forms questionnaire. All participants were aged 18 years or older, so parental or guardian consent was not required.

**Comment 3:** *Provide an amended Funding Statement declaring all sources of support, including the sentence "There was no additional external funding received for this study."*

**Response 3:** The amended Funding Statement is: "This work was supported by Prince Sattam Bin Abdulaziz University, project number PSAU/2025/01/38872, awarded to A.M.A. There was no additional external funding received for this study." It is also included in our cover letter.

**Comment 4:** *Remove funding-related text from the manuscript.*

**Response 4:** We deleted the Funding section, which contained the acknowledgment of Prince Sattam bin Abdulaziz University. The Acknowledgments now only thank the participants. The amended Funding Statement appears in the cover letter.

**Comment 5:** *Evaluate any reviewer-recommended citations.*

**Response 5:** Neither reviewer recommended specific publications, so no citations were added on this basis.

**Comment 7:** *Review the reference list for completeness and retracted papers.*

**Response 7:** we checked the reference list for completeness and for retracted articles and found none. No references were added or removed in this revision.

# Academic Editor

**Comment 1:** *Please address the comments provided by the reviewers.*

**Response 1:** All reviewer comments are addressed point by point below.

# Reviewer #1

**Comment 1:** *The "first empirical evidence" claim is difficult to defend, because an association between academic entitlement and the impostor phenomenon has already been documented in graduate students. Clarify the scope of the literature search and temper the claim.*

**Response 1:** We agree and have tempered the claim. The Conclusion now reads "To our knowledge, the present study is the first to test the impostor phenomenon as a mediator between academic entitlement and well-being among undergraduates in a non-Western (Egyptian) context." The phrase "absolute lack of empirical evidence" in the Introduction was replaced. The Introduction now acknowledges the graduate-student association reported by Gibson-Beverly and Schwartz [71] and states the gap more narrowly.

In the Discussion we now describe [71] as empirical (not conceptual) work, and state that our findings extend it to Egyptian undergraduates and to well-being outcomes. The cover letter was revised in the same way.

**Comment 2:** *It is unclear whether mediation was tested for each well-being dimension or only for a composite score; claims about psychological, physical, and relational well-being are therefore unwarranted.*

**Response 2:** Mediation was tested once, using the composite BBC-WBS score. We have made this explicit by adding a sentence to the Mediation analysis subsection and to the Results summary. We removed all claims that the indirect effect operates across psychological, physical, and relational well-being, in the Abstract, Introduction, Results, Discussion, and Conclusion. These sections now refer to "overall well-being (composite score)."

The Limitations and Future research sections now state that the indirect effect may differ across dimensions and recommend testing each dimension separately.

**Comment 3:** *Generalizability and cultural context: acknowledge limited generalizability, discuss cultural factors, avoid overgeneralizing recommendations, and report validation and measurement invariance of the instruments.*

**Response 3:** We addressed each part. (a) The Limitations now state that findings may not generalize beyond the Egyptian context, including to individualistic Western settings. (b) A new Discussion paragraph considers how collectivist factors, such as family expectations, social reputation, and fear of shaming one's family, might shape entitlement and impostor feelings. We label these interpretations as speculative because they were not measured. (c) The practical recommendations in the Abstract, Discussion, and Conclusion now refer to Egyptian and comparable contexts, and are described as tentative.

We added a paragraph to the Measures subsection explaining that the AES-28 was developed and validated for Egyptian students, while the CIPS-10 and BBC-WBS were translated, back-translated, and evaluated with CFA and reliability analyses. Measurement invariance was not tested, and we now list this as a limitation.

**Comment 4:** *The note to Table 2 does not explain the academic entitlement subscales.*

**Response 4:** The note now defines all abbreviations (ER, EE, CM, COLE, RME, AES-28, CIPS-10, PWB, PHWB, REL, BBC-WBS) and gives item counts for the subscales.

**Comment 5:** *Labels for paths a, b, and c′ are missing in Figure 1; label all paths, show direction, and explain them in the caption.*

**Response 5:** We redrew Figure 1. It now labels paths a, b, and c′ with their standardized coefficients and shows the total effect c and the indirect effect (a × b). Arrows indicate direction. A note below the caption explains each path. The values match Tables 4 and 5.

**Comment 6:** *Locus of control is a relevant variable that was not considered; acknowledge this limitation and discuss how controlling for it might alter the results.*

**Response 6:** We added this as a limitation. The text notes that external locus of control is linked to both academic entitlement and the impostor phenomenon, so the observed associations may be partly confounded. It also states that adjusting for it could attenuate the paths and the indirect effect. Future research now includes locus of control as a covariate or competing mediator.

# Reviewer #2

**Comment 1:** *The Ethics Statement should appear at the beginning of the Methods, before the Study Design, and should state the type of consent or whether consent was waived.*

**Response 1:** We moved the Ethics statement to the start of the Materials and methods section, before Study design. It now describes the IRB approval and the consent procedure (see Response to Journal Requirement 2).

**Comment 2:** *Data availability (answered "No" in the review form).*

**Response 2:** We revised the Data Availability Statement to name a non-author institutional contact. [https://doi.org/10.5281/zenodo.23258159]

We thank the Editor and Reviewers for helping us improve the manuscript.

Sincerely,
Mohammed Rahmath (Corresponding Author), on behalf of all co-authors
