# Supplementary material for: Academic entitlement and student well-being: The mediating role of the impostor phenomenon
Source: PLoS One. 2026 Nov 4;21(11):e0362076. doi: 10.1371/journal.pone.0362076 (PMC13734581; doi:10.1371/journal.pone.0362076)
Supplement: Peer review history

# Peer review: Decision Letter 0

**DOI:** 10.1371/journal.pone.0362076.r001

**Authors:** Marko Joksimovic

7 Oct 2026

Dear Dr. Nemt-allah,

Thank you for submitting your manuscript to PLOS One. After careful consideration, we feel that it has merit but does not fully meet PLOS One’s publication criteria as it currently stands. Therefore, we invite you to submit a revised version of the manuscript that addresses the points raised during the review process.

Please submit your revised manuscript by Nov 05 2026 11:59PM. If you will need more time than this to complete your revisions, please reply to this message or contact the journal office at plosone@plos.org. When you're ready to submit your revision, log on to https://www.editorialmanager.com/pone/ and select the 'Submissions Needing Revision' folder to locate your manuscript file.

- A letter that responds to each point raised by the academic editor and reviewer(s). You should upload this letter as a separate file labeled 'Response to Reviewers'.
- A marked-up copy of your manuscript that highlights changes made to the original version. You should upload this as a separate file labeled 'Revised Manuscript with Track Changes'.
- An unmarked version of your revised paper without tracked changes. You should upload this as a separate file labeled 'Manuscript'.

If you would like to make changes to your financial disclosure, please include your updated statement in your cover letter. Guidelines for resubmitting your figure files are available below the reviewer comments at the end of this letter.

If applicable, we recommend that you deposit your laboratory protocols in protocols.io to enhance the reproducibility of your results. Protocols.io assigns your protocol its own identifier (DOI) so that it can be cited independently in the future. For instructions see: https://journals.plos.org/plosone/s/submission-guidelines#loc-laboratory-protocols. Additionally, PLOS One offers an option for publishing peer-reviewed Lab Protocol articles, which describe protocols hosted on protocols.io. Read more information on sharing protocols at https://plos.org/protocols?utm_medium=editorial-email&utm_source=authorletters&utm_campaign=protocols.

As the corresponding author, your ORCID iD is verified in the submission system and will appear in the published article. PLOS supports the use of ORCID, and we encourage all coauthors to register for an ORCID iD and use it as well. Please encourage your coauthors to verify their ORCID iD within the submission system before final acceptance, as unverified ORCID iDs will not appear in the published article. *Only*  the individual author can complete the verification step; PLOS staff *cannot*  verify ORCID iDs on behalf of authors.

We look forward to receiving your revised manuscript.

Kind regards,

Marko Joksimovic

Academic Editor

PLOS One

Journal Requirements:

When submitting your revision, we need you to address these additional requirements.

1. Please ensure that your manuscript meets PLOS ONE's style requirements, including those for file naming. The PLOS ONE style templates can be found at

https://journals.plos.org/plosone/s/file?id=wjVg/PLOSOne_formatting_sample_main_body.pdf and

https://journals.plos.org/plosone/s/file?id=ba62/PLOSOne_formatting_sample_title_authors_affiliations.pdf.

2. Please provide details regarding participant consent. In the ethics statement in the Methods and online submission information, please ensure that you have specified (1) whether consent was informed and (2) what type you obtained (for instance, written or verbal, and if verbal, how it was documented and witnessed). If your study included minors, state whether you obtained consent from parents or guardians. If the need for consent was waived by the ethics committee, please include this information.

If you are reporting a retrospective study of medical records or archived samples, please ensure that you have discussed whether all data were fully anonymized before you accessed them and/or whether the IRB or ethics committee waived the requirement for informed consent. If patients provided informed written consent to have data from their medical records used in research, please include this information

3.Thank you for stating in your Funding Statement:

“This work was supported by Prince Sattam Bin Abdulaziz University, project number PSAU/2025/01/38872, awarded to A.M.A. (Funding acquisition). “

Please provide an amended statement that declares *all* the funding or sources of support (whether external or internal to your organization) received during this study, as detailed online in our guide for authors at http://journals.plos.org/plosone/s/submit-now.  Please also include the statement “There was no additional external funding received for this study.” in your updated Funding Statement.

Please include your amended Funding Statement within your cover letter. We will change the online submission form on your behalf.

4. In the online submission form you indicate that your data is not available for proprietary reasons and have provided a contact point for accessing this data. Please note that your current contact point is a co-author on this manuscript. According to our Data Policy, the contact point must not be an author on the manuscript and must be an institutional contact, ideally not an individual. Please revise your data statement to a non-author institutional point of contact, such as a data access or ethics committee, and send this to us via return email. Please also include contact information for the third party organization, and please include the full citation of where the data can be found.

5. Thank you for stating the following in your manuscript:

“The authors extend their appreciation to Prince Sattam bin Abdulaziz University for funding this research work through the project number (PSAU/ 2025/01/38872).”

We note that you have provided additional information within the manuscript that is not currently declared in your Funding Statement. Please note that funding information should not appear in the Acknowledgments section or other areas of your manuscript. We will only publish funding information present in the Funding Statement section of the online submission form.

Please remove any funding-related text from the manuscript and let us know how you would like to update your Funding Statement. Currently, your Funding Statement reads as follows:

“This work was supported by Prince Sattam Bin Abdulaziz University, project number PSAU/2025/01/38872, awarded to A.M.A. (Funding acquisition).”

Please include your amended statements within your cover letter; we will change the online submission form on your behalf.

6. If the reviewer comments include a recommendation to cite specific previously published works, please review and evaluate these publications to determine whether they are relevant and should be cited. There is no requirement to cite these works unless the editor has indicated otherwise.

7. Please review your reference list to ensure that it is complete and correct. If you have cited papers that have been retracted, please include the rationale for doing so in the manuscript text, or remove these references and replace them with relevant current references. Any changes to the reference list should be mentioned in the rebuttal letter that accompanies your revised manuscript. If you need to cite a retracted article, indicate the article’s retracted status in the References list and also include a citation and full reference for the retraction notice.

Additional Editor Comments (if provided):

Dear Authors,

Please address the comments provided by the reviewers.

Reviewer 1:

Thank you for the opportunity to review the manuscript “Academic Entitlement and Student Well-Being: The Mediating Role of the Impostor Phenomenon”. The topic is timely, and the Egyptian sample adds valuable non-Western evidence to the literature. Below are several comments aimed at improving the paper:

1. The "First Empirical Evidence" Claim

The authors assert that the present study "provides the first empirical evidence that the impostor phenomenon mediates the relationship between academic entitlement and student well-being." This is a strong novelty claim and requires careful scrutiny. The literature already documents an association between academic entitlement and the impostor phenomenon in graduate student populations, even if the full mediation model with well-being as an outcome has not been tested. The authors should clarify the scope of their literature search and, if prior related work exists, temper the claim accordingly (e.g., "first in a non-Western context" or "first to test well-being as an outcome"). As it stands, the claim of absolute priority is difficult to defend and should either be substantiated or revised.

2. "Holistic Functioning" and the Three Dimensions of Well-Being

The conclusion claims that academic entitlement undermines students' "psychological, physical, and relational well-being" and refers to "holistic functioning." However, it is unclear whether the mediation model was tested separately for each of the three well-being dimensions or only for a composite well-being score. If a single composite score was used, the claim that the indirect effect operates uniformly across all three dimensions is not warranted. The mediating role of the impostor phenomenon may be strong for psychological well-being but weak or non-significant for physical or relational well-being.

3. Generalizability and Cultural Context

The conclusion speaks of "universities" in general and calls upon them to address entitled attitudes and impostor-related cognitions. However, the sample consists exclusively of Egyptian university students. This raises significant concerns about generalizability. In collectivist cultures, academic entitlement may manifest differently than in individualistic Western cultures, for example, it may be tied more closely to family expectations or social reputation rather than individual self-interest. Similarly, the impostor phenomenon in collectivist contexts may be driven more by fear of bringing shame to one's family or community than by individual self-doubt. The authors should (a) acknowledge that their findings may not generalize beyond the Egyptian context, (b) discuss how cultural factors might shape the relationships observed, and (c) avoid overgeneralizing their practical recommendations to "universities" as a universal category. Ideally, the manuscript should also report whether the measurement instruments used were validated for the Egyptian context and whether measurement invariance was established.

4. Insufficient Description of Academic Entitlement Subscales in Table 2

In Table 2, which presents descriptive statistics, the table note does not provide any explanation of the academic entitlement subscales.

5. Missing Path Labels in Figure 1

In Figure 1, the labels for paths a, b, and c′ are not visible. Without these labels, the reader cannot easily map the figure onto the hypotheses stated in the introduction or the results reported in the text. This creates unnecessary confusion and undermines the clarity of the mediation model. The authors should revise Figure 1 to explicitly label all paths (a, b, c, and c′), include the direction of arrows, and ensure that the figure caption explains what each path represents. Consistency between the figure, the hypotheses, and the results tables is essential.

6. Locus of Control as an Unconsidered Variable

It appears that locus of control could have been a relevant variable in this study but was not included in the model. Locus of control is theoretically and empirically linked to both academic entitlement (external locus of control may foster entitled expectations) and the impostor phenomenon. At a minimum, the authors should acknowledge this limitation in the limitations section and discuss how controlling for locus of control might have altered the observed relationships.

Reviewer 2:

The paper is well written, I hope my comments will help you further to improve it.

Materials and Methods:

The Ethics Statement is usually presented at the beginning of the Methods section, before the Study Design. In this section, in addition to providing the IRB approval details, please mention the type of consent used for the current study or indicate whether the study was exempt from the consent requirement.

[Note: HTML markup is below. Please do not edit.]

Reviewers' comments:

Reviewer's Responses to Questions

**Comments to the Author**

1. Is the manuscript technically sound, and do the data support the conclusions?

Reviewer #1: Yes

Reviewer #2: Yes

**********

2. Has the statistical analysis been performed appropriately and rigorously?

Reviewer #1: Yes

Reviewer #2: Yes

**********

3. Have the authors made all data underlying the findings in their manuscript fully available?

The PLOS Data policy

Reviewer #1: Yes

Reviewer #2: No

**********

4. Is the manuscript presented in an intelligible fashion and written in standard English?

Reviewer #1: Yes

Reviewer #2: Yes

**********

Reviewer #1: Thank you for the opportunity to review the manuscript “Academic Entitlement and Student Well-Being: The Mediating Role of the Impostor Phenomenon”. The topic is timely, and the Egyptian sample adds valuable non-Western evidence to the literature. Below are several comments aimed at improving the paper:

1. The "First Empirical Evidence" Claim

The authors assert that the present study "provides the first empirical evidence that the impostor phenomenon mediates the relationship between academic entitlement and student well-being." This is a strong novelty claim and requires careful scrutiny. The literature already documents an association between academic entitlement and the impostor phenomenon in graduate student populations, even if the full mediation model with well-being as an outcome has not been tested. The authors should clarify the scope of their literature search and, if prior related work exists, temper the claim accordingly (e.g., "first in a non-Western context" or "first to test well-being as an outcome"). As it stands, the claim of absolute priority is difficult to defend and should either be substantiated or revised.

2. "Holistic Functioning" and the Three Dimensions of Well-Being

The conclusion claims that academic entitlement undermines students' "psychological, physical, and relational well-being" and refers to "holistic functioning." However, it is unclear whether the mediation model was tested separately for each of the three well-being dimensions or only for a composite well-being score. If a single composite score was used, the claim that the indirect effect operates uniformly across all three dimensions is not warranted. The mediating role of the impostor phenomenon may be strong for psychological well-being but weak or non-significant for physical or relational well-being.

3. Generalizability and Cultural Context

The conclusion speaks of "universities" in general and calls upon them to address entitled attitudes and impostor-related cognitions. However, the sample consists exclusively of Egyptian university students. This raises significant concerns about generalizability. In collectivist cultures, academic entitlement may manifest differently than in individualistic Western cultures — for example, it may be tied more closely to family expectations or social reputation rather than individual self-interest. Similarly, the impostor phenomenon in collectivist contexts may be driven more by fear of bringing shame to one's family or community than by individual self-doubt. The authors should (a) acknowledge that their findings may not generalize beyond the Egyptian context, (b) discuss how cultural factors might shape the relationships observed, and (c) avoid overgeneralizing their practical recommendations to "universities" as a universal category. Ideally, the manuscript should also report whether the measurement instruments used were validated for the Egyptian context and whether measurement invariance was established.

4. Insufficient Description of Academic Entitlement Subscales in Table 2

In Table 2, which presents descriptive statistics, the table note does not provide any explanation of the academic entitlement subscales.

5. Missing Path Labels in Figure 1

In Figure 1, the labels for paths a, b, and c′ are not visible. Without these labels, the reader cannot easily map the figure onto the hypotheses stated in the introduction or the results reported in the text. This creates unnecessary confusion and undermines the clarity of the mediation model. The authors should revise Figure 1 to explicitly label all paths (a, b, c, and c′), include the direction of arrows, and ensure that the figure caption explains what each path represents. Consistency between the figure, the hypotheses, and the results tables is essential.

6. Locus of Control as an Unconsidered Variable

It appears that locus of control could have been a relevant variable in this study but was not included in the model. Locus of control is theoretically and empirically linked to both academic entitlement (external locus of control may foster entitled expectations) and the impostor phenomenon. At a minimum, the authors should acknowledge this limitation in the limitations section and discuss how controlling for locus of control might have altered the observed relationships.

Reviewer #2: Dear Authors,

The paper is well written, I hope my comments will help you further to improve it.

Materials and Methods:

The Ethics Statement is usually presented at the beginning of the Methods section, before the Study Design. In this section, in addition to providing the IRB approval details, please mention the type of consent used for the current study or indicate whether the study was exempt from the consent requirement.

**********

what does this mean?). If published, this will include your full peer review and any attached files.

If you choose “no”, your identity will remain anonymous but your review may still be made public.

**Do you want your identity to be public for this peer review?**  For information about this choice, including consent withdrawal, please see our Privacy Policy

Reviewer #1: No

Reviewer #2: No

**********

[NOTE: If reviewer comments were submitted as an attachment file, they will be attached to this email and accessible via the submission site. Please log into your account, locate the manuscript record, and check for the action link "View Attachments". If this link does not appear, there are no attachment files.]

To ensure your figures meet our technical requirements, please review our figure guidelines: https://journals.plos.org/plosone/s/figures

You may also use PLOS’s free figure tool, NAAS, to help you prepare publication quality figures: https://journals.plos.org/plosone/s/figures#loc-tools-for-figure-preparation.

NAAS will assess whether your figures meet our technical requirements by comparing each figure against our figure specifications.

Attachment Submitted filename: Manuscript Title- plos one.docx

# Author comment: Author response to Decision Letter 1

**DOI:** 10.1371/journal.pone.0362076.r002

11 Oct 2026

Reviewer #1

Comment 1: The "first empirical evidence" claim is difficult to defend, because an association between academic entitlement and the impostor phenomenon has already been documented in graduate students. Clarify the scope of the literature search and temper the claim.

Response 1: We agree and have tempered the claim. The Conclusion now reads "To our knowledge, the present study is the first to test the impostor phenomenon as a mediator between academic entitlement and well-being among undergraduates in a non-Western (Egyptian) context." The phrase "absolute lack of empirical evidence" in the Introduction was replaced. The Introduction now acknowledges the graduate-student association reported by Gibson-Beverly and Schwartz [71] and states the gap more narrowly.

In the Discussion we now describe [71] as empirical (not conceptual) work, and state that our findings extend it to Egyptian undergraduates and to well-being outcomes. The cover letter was revised in the same way.

Comment 2: It is unclear whether mediation was tested for each well-being dimension or only for a composite score; claims about psychological, physical, and relational well-being are therefore unwarranted.

Response 2: Mediation was tested once, using the composite BBC-WBS score. We have made this explicit by adding a sentence to the Mediation analysis subsection and to the Results summary. We removed all claims that the indirect effect operates across psychological, physical, and relational well-being, in the Abstract, Introduction, Results, Discussion, and Conclusion. These sections now refer to "overall well-being (composite score)."

The Limitations and Future research sections now state that the indirect effect may differ across dimensions and recommend testing each dimension separately.

Comment 3: Generalizability and cultural context: acknowledge limited generalizability, discuss cultural factors, avoid overgeneralizing recommendations, and report validation and measurement invariance of the instruments.

Response 3: We addressed each part. (a) The Limitations now state that findings may not generalize beyond the Egyptian context, including to individualistic Western settings. (b) A new Discussion paragraph considers how collectivist factors, such as family expectations, social reputation, and fear of shaming one's family, might shape entitlement and impostor feelings. We label these interpretations as speculative because they were not measured. (c) The practical recommendations in the Abstract, Discussion, and Conclusion now refer to Egyptian and comparable contexts, and are described as tentative.

We added a paragraph to the Measures subsection explaining that the AES-28 was developed and validated for Egyptian students, while the CIPS-10 and BBC-WBS were translated, back-translated, and evaluated with CFA and reliability analyses. Measurement invariance was not tested, and we now list this as a limitation.

Comment 4: The note to Table 2 does not explain the academic entitlement subscales.

Response 4: The note now defines all abbreviations (ER, EE, CM, COLE, RME, AES-28, CIPS-10, PWB, PHWB, REL, BBC-WBS) and gives item counts for the subscales.

Comment 5: Labels for paths a, b, and c′ are missing in Figure 1; label all paths, show direction, and explain them in the caption.

Response 5: We redrew Figure 1. It now labels paths a, b, and c′ with their standardized coefficients and shows the total effect c and the indirect effect (a × b). Arrows indicate direction. A note below the caption explains each path. The values match Tables 4 and 5.

Comment 6: Locus of control is a relevant variable that was not considered; acknowledge this limitation and discuss how controlling for it might alter the results.

Response 6: We added this as a limitation. The text notes that external locus of control is linked to both academic entitlement and the impostor phenomenon, so the observed associations may be partly confounded. It also states that adjusting for it could attenuate the paths and the indirect effect. Future research now includes locus of control as a covariate or competing mediator.

Reviewer #2

Comment 1: The Ethics Statement should appear at the beginning of the Methods, before the Study Design, and should state the type of consent or whether consent was waived.

Response 1: We moved the Ethics statement to the start of the Materials and methods section, before Study design. It now describes the IRB approval and the consent procedure (see Response to Journal Requirement 2).

Comment 2: Data availability (answered "No" in the review form).

Response 2: We revised the Data Availability Statement to name a non-author institutional contact. [https://doi.org/10.5281/zenodo.23258159]

We thank the Editor and Reviewers for helping us improve the manuscript.

Sincerely,

Mohammed Rahmath (Corresponding Author), on behalf of all co-authors

Attachment Submitted filename: Response_to_Reviewers.docx

# Editor report: Decision Letter 1

**DOI:** 10.1371/journal.pone.0362076.r003

**Authors:** Marko Joksimovic

20 Oct 2026

After reviewing the revised manuscript and the authors’ responses to the comments of both reviewers, I conclude that the authors have adequately addressed the main concerns and implemented the necessary revisions. The manuscript has been improved in terms of clarifying its research contribution, presenting and interpreting the results, discussing the study’s limitations, defining terms in the tables, illustrating the mediation model, and reporting the ethical procedures. The remaining observations mainly concern final checks of consistency and technical details and do not constitute obstacles to publication. Therefore, I recommend that the manuscript be accepted for publication.

# Editor report: Acceptance letter

**DOI:** 10.1371/journal.pone.0362076.r004

**Authors:** Marko Joksimovic

PONE-D-26-37507R1

PLOS One

Dear Dr. Nemt-allah,

I'm pleased to inform you that your manuscript has been deemed suitable for publication in PLOS One. Congratulations! Your manuscript is now being handed over to our production team.

At this stage, our production department will prepare your paper for publication. This includes ensuring the following:

* All references, tables, and figures are properly cited

* All relevant supporting information is included in the manuscript submission,

* There are no issues that prevent the paper from being properly typeset

You will receive further instructions from the production team, including instructions on how to review your proof when it is ready. Please keep in mind that we are working through a large volume of accepted articles, so please give us a few days to review your paper and let you know the next and final steps.

Lastly, if your institution or institutions have a press office, please let them know about your upcoming paper now to help maximize its impact. If they'll be preparing press materials, please inform our press team within the next 48 hours. Your manuscript will remain under strict press embargo until 2 pm Eastern Time on the date of publication. For more information, please contact onepress@plos.org.

You will receive an invoice from PLOS for your publication fee after your manuscript has reached the completed accept phase. If you receive an email requesting payment before acceptance or for any other service, this may be a phishing scheme. Learn how to identify phishing emails and protect your accounts at https://explore.plos.org/phishing.

If we can help with anything else, please email us at customercare@plos.org.

Thank you for submitting your work to PLOS One and supporting open access.

Kind regards,

PLOS One Editorial Office Staff

on behalf of

Dr. Marko Joksimovic

Academic Editor

PLOS One
